# Breaking Barriers: The Role of the Bone Marrow Microenvironment in Multiple Myeloma Progression

**DOI:** 10.3390/ijms26157301

**Published:** 2025-07-28

**Authors:** Aleksandra Agafonova, Chiara Prinzi, Angela Trovato Salinaro, Caterina Ledda, Alessia Cosentino, Maria Teresa Cambria, Carmelina Daniela Anfuso, Gabriella Lupo

**Affiliations:** 1 Department of Biomedical and Biotechnological Sciences, Section of Medical Biochemistry, University of Catania, 95123 Catania, Italy; aleksandraaagafonova@gmail.com (A.A.); chiara27chiara@gmail.com (C.P.); alessia1993@hotmail.it (A.C.); gabriella.lupo@unict.it (G.L.); 2 Department of Biomedical and Biotechnological Sciences, Section of Clinical Biochemistry, University of Catania, 95123 Catania, Italy; angela.trovato@unict.it; 3 Department of Clinical and Experimental Medicine, Occupational Medicine, University of Catania, 95124 Catania, Italy; caterina.ledda@unict.it

**Keywords:** multiple myeloma, bone marrow barrier, bone marrow endothelial cells, bone marrow stromal cells

## Abstract

Multiple myeloma (MM) is an incurable malignancy characterized by the proliferation of abnormal plasma cells within the bone marrow, followed by potential dissemination to extramedullary sites. The bone marrow barrier (BMB) plays a pivotal role in plasma cell homing and disease progression. Bone marrow endothelial cells (BMECs) and bone marrow stromal cells (BMSCs), through their interactions with MM cells, secrete adhesion molecules, angiogenic cytokines, anti-apoptotic factors, and growth-promoting signals that support MM cell survival and proliferation. This review examines the components of the BMB and the major pathways involved in MM pathogenesis. Targeting the interactions between MM cells and the BMB may offer novel therapeutic opportunities.

## 1. Bone Marrow Barrier

The bone marrow barrier (BMB) refers to the specialized vascular structures, particularly the sinusoidal endothelium, that regulate the entry and exit of cells, molecules, and substances between the bone marrow and the systemic circulation. It plays a crucial role in maintaining the integrity and functionality of the bone marrow microenvironment, protecting it against pathogens, toxins, and harmful agents while supporting hematopoiesis, which is the process of blood cell formation [1,2].

The BMB consists of bone marrow endothelial cells (BMECs), adventitial cells, and the basement membrane, together forming the blood sinus wall [2]. BMECs regulate the selective passage of cells and substances, with intercellular tight junctions and a capacity for forming transcellular pores [3,4]. Adventitial cells cover the external surface of the sinus wall and extend processes to form a supportive reticular network. Although the adventitial layer is incomplete, it may contribute to regulating cellular egress by modulating vascular fenestrations [5]. The basement membrane, composed of laminin, proteoglycans, and collagen, provides structural support and regulates signaling within the marrow microenvironment, also facilitating the egress of mature blood cells [6].

Perivascular cells, including pericytes, surround the endothelial cells and are essential for vessel stability and regulation of blood flow [7]. Perisinusoidal macrophages are positioned near the sinusoids and are involved in the phagocytosis of defective cells and erythrocyte nuclei [8]. Bone marrow stromal cells (BMSCs), also known as mesenchymal stromal cells, are multipotent cells capable of differentiating into adipocytes, osteoblasts, and chondrocytes. They contribute to bone remodeling, repair, and secrete hematopoietic support factors [9].

The sinusoidal wall is thin, composed of a continuous monolayer of endothelial cells. Blood cells and lymphocytes can cross the BMB primarily via transcellular migration, traversing the cytoplasm of the sinusoidal endothelial cells [10,11]. Mature blood cells form intracellular pores in order to migrate, although mechanisms differ between cell types [12]. Red blood cells deform in order to pass through endothelial gaps, while leukocytes adhere to the sinusoidal wall and subsequently transmigrate. Under physiological conditions, immature blood cells are typically retained within the marrow [13].

## 2. Abnormal Bone Marrow Barrier

The BMB plays a key role in hematopoietic homeostasis, and its integrity can be compromised in both neoplastic and non-neoplastic conditions.

Different diseases, such as multiple myeloma (MM), leukemia, and bone metastases from other types of cancer, could disrupt the sinus wall, and this may allow a large number of immature cells to pass through the sinusoidal cells. It has been shown that tumor cells, like Acute Myeloid Leukemia (AML) cells, could secrete pro-inflammatory cytokines like interleukin-1 (IL-1) and tumor necrosis factor (TNF-α), which induce the expression of adhesion molecules, such as intercellular cell adhesion molecule (ICAM), vascular cell adhesion molecule-1 (VCAM-1), and E-selectin by BMECs [14,15,16,17,18]. The expression of these adhesion molecules promotes the adhesion of tumor cells to the endothelium and activates pro-survival signals. Adhesion-mediated activation of pro-survival pathways protects AML cells from apoptosis and makes them less sensitive to chemotherapy, contributing to their resistance to targeted therapies and immune surveillance [19]. In particular, the interaction of E-selectin with AML-blasts allows them to hide into the protective bone marrow niche, mediating cell survival through the activation of AKT/NF-κB/mTOR pathways [20,21]. The interaction between VCAM-1 and very late antigen-4 (VLA-4) activates NF-kB in BMSCs and leukemia cells, promoting chemoresistance [22,23]. Drug resistance is also induced by the interaction of VLA-4 expressed on leukemic cells with fibronectin on BMSCs, activating the PI-3K/AKT/Bcl-2 signaling pathway [24,25].

Tumor cells, including hematological malignancies like AML and MM, can profoundly remodel the bone marrow microenvironment, particularly the BMB, by promoting angiogenesis. Angiogenesis, the process of forming new blood vessels from pre-existing vasculature, is a hallmark of cancer progression. Tumor cells exploit this process to secure nutrients, oxygen, and pathways for dissemination [26].

Vascular endothelial growth factor (VEGF) is the main pro-angiogenic factor in AML. Leukemic cells overexpress VEGF and its receptors [27]. VEGF is secreted into the bone marrow microenvironment, where it acts in both autocrine and paracrine manners. In paracrine signaling, VEGF activates BMECs, promoting angiogenesis and the expansion of the vascular network. In autocrine signaling, VEGF binds VEGFRs on the leukemia cells, enhancing their survival and proliferation through NF-kB, Akt, Erk, and Bcl-2 signaling pathways [28,29,30,31,32].

Angiopoietins, particularly Angiopoietin-1 (Ang-1) and Angiopoietin-2 (Ang-2), are critical regulators of vascular remodeling and angiogenesis, including leukemia-induced angiogenesis. Their interaction with the Tie2 receptor on endothelial cells determines vascular stability, maturation, and permeability, playing a dual role in vascular homeostasis and tumor progression. Ang-1 stabilizes blood vessels by promoting the interaction between endothelial cells and pericytes. Ang-2 acts as an antagonist of Ang-1 and is often overexpressed in leukemia, disrupting vascular stability and promoting angiogenesis [33,34].

Remodeling is associated with disruption to the sinus wall, increasing vascular permeability and trans-endothelial cell migration, due to the higher production of nitric oxide (NO) by BMECs [35]. Tumor cells and inflammatory cytokines such as TNF-α and IL-1 stimulate BMECs to produce higher amounts of NO via the upregulation of inducible nitric oxide synthase (iNOS). Furthermore, VEGF activates endothelial NOS (eNOS) to produce NO through the PI3K/AKT signaling pathway [36]. NO disrupts endothelial junctions, increasing endothelial permeability. This permeability provides leukemia cells with greater access to systemic circulation and facilitates metastasis [37].

Tumor cells and activated BMECs secrete matrix metalloproteinases (MMPs), such as MMP-9, which degrade the extracellular matrix (ECM), facilitating endothelial cell migration [38].

Newly formed blood vessels are often immature and leaky, leading to altered barrier function. This facilitates tumor cell dissemination into the bloodstream, promoting metastasis. In addition, tumor-induced disruption of the sinusoidal walls and vascular permeability also lead to altered oxygen diffusion, creating localized hypoxic regions, which may contribute to disease progression [39,40,41].

Non-neoplastic diseases could also affect BMB. Hypertension, atherosclerosis, and acute myocardial infarction may impair endothelial cells and promote vascular permeability and angiogenesis, leading to leukocytosis [42]. Chronic inflammatory diseases, like rheumatoid arthritis, are associated with the higher production of pro-inflammatory factors, including IL-1, IL-6, and TNF-α, which leads to increased permeability of BMB and the enhanced egress of monocytes from the bone marrow [43,44].

## 3. Multiple Myeloma

MM is a hematological malignancy characterized by the clonal proliferation of transformed plasma cells that preferentially accumulate in the bone marrow. The transformation of plasma cells into malignant cells appears to involve three main mechanisms. First, VDJH recombination—the rearrangement of variable (V), diversity (D), and joining (J) segments of the immunoglobulin (*Ig*) gene—occurs in precursor B cells within the bone marrow. Second, errors in somatic hypermutation may arise during plasma cell generation in germinal centers. Third, aberrant class switch recombination in post-germinal center plasma cells can lead to dysregulated *Ig* heavy chain (*IgH*) expression, promoting bone marrow homing [45,46].

Among these mechanisms, translocations involving the *IgH* gene locus at 14q32 are particularly significant, occurring in up to 70% of MM cases and playing a central role in pathogenesis by altering the expression of key oncogenes [47,48]. The most common translocation, t(11;14)(q13;q32), results in overexpression of cyclin D1 (*CCND1*) and is observed in approximately 20% of patients [49,50]. Similarly, the t(4;14)(p16;q32) translocation, found in about 15% of cases, leads to upregulation of *FGFR3* and the formation of an *IgH-MMSET* fusion transcript [27,51]. Another noteworthy translocation, t(14;16)(q32;q23), occurs in 5–10% of cases and drives c-MAF overexpression, which in turn activates cyclin D2, integrin β, and chemokine receptor type 1 (*CCR1)*, collectively promoting MM cell proliferation [52,53].

For instance, translocations at 8q24 dysregulate *MYC*, driving uncontrolled proliferation, the development of extramedullary MM (EMM), and poor clinical outcomes [48]. While *MYC* translocations are detected in roughly 15% of newly diagnosed patients, *MYC* overexpression is present in about 40%, suggesting that the two are not always directly linked [54,55]. The gain of 1q is associated with the dysregulation of genes such as *BCL9*, *CKS1B*, and *MCL-1*, and correlates with disease progression and poorer prognosis [56]. Meanwhile, deletion of 17p, which harbors the *TP53* tumor suppressor gene, impairs DNA repair, cell cycle regulation, and apoptosis—leading to more aggressive disease and a higher likelihood of EMM [57].

Clinically, MM often evolves from monoclonal gammopathy of undetermined significance (MGUS), an asymptomatic premalignant condition. MGUS can progress to an intermediate stage called smoldering multiple myeloma (SMM) before developing into full-blown MM [58,59,60]. Common clinical features include hypercalcemia, renal failure, anemia, and bone lesions—collectively referred to by the acronym CRAB—along with other complications [61,62].

## 4. Multiple Myeloma and Bone Marrow Barrier

EMM represents an aggressive form of MM, particularly during relapse, in which a clone or subclone of malignant plasma cells escapes the BMB and proliferates in extramedullary sites. EMM is typically classified into two types: bone-related EMM and extraosseous EMM. The former results from cortical bone destruction and local tumor extension, while the latter arises via hematogenous dissemination, leading to the development of soft-tissue plasmacytomas [63]. At diagnosis, EMM frequently involves skin and soft tissues; during relapse, it may affect a broader range of organs, including the liver, pleura, kidneys, lymph nodes, breast, spleen, pericardium, and central nervous system (CNS). CNS involvement, though rare, is particularly severe, with plasma cells infiltrating the leptomeninges, brain parenchyma, or cerebrospinal fluid (CSF), leading to the destruction of arachnoid trabeculae [64,65].

The ability of malignant plasma cells to overcome the BMB is mediated by several mechanisms. These include the downregulation of adhesion molecules, reduced expression of chemokine receptors, increased angiogenesis, and decreased levels of the tetraspanins CD81 and CD82. Additionally, overexpression of heparanase-1 and the accumulation of genetic mutations further facilitate detachment from the bone marrow microenvironment, supporting systemic dissemination [66,67,68,69].

MMPs, particularly MMP-2 and MMP-9, also play a central role by degrading ECM components such as collagen and gelatin. These enzymes are often overexpressed in MM, contributing both to bone lesion formation and to the extramedullary migration of plasma cells [70].

EMM is associated with significantly poorer prognosis compared to intramedullary MM. In EMM, MM cells reduce their dependence on the bone marrow niche, due to a lower expression of adhesion molecules and chemokine receptors, and acquire enhanced migratory and invasive capabilities [66]. These features contribute to disease dissemination and to a reduced efficacy of therapies that primarily target tumor–microenvironment interactions. As a result, MM cells are often resistant to immunomodulatory drugs and targeted therapies, leading to increased treatment failure. Moreover, EMM is more prone to relapse, due to the acquisition of a more aggressive and genetically unstable phenotype, and resistance to apoptosis. Clinically, EMM is more difficult to monitor, complicating the early detection of relapses or residual disease [71,72].

Understanding the interactions between myeloma cells and the bone marrow niche, including the structural and molecular components of the BMB, is crucial for the development of novel therapeutic strategies aimed at preventing dissemination and improving patient outcomes.

### 4.1. Homing Pathways

Cyclophilin A (CyPA), secreted by BMECs, acts as a homing factor by binding to the CD147 receptor on MM cells. This interaction promotes MM cell colonization, proliferation within the bone marrow, and drug resistance. Inhibiting CyPA via small interfering RNA (siRNA) has been proposed as a potential strategy to prevent MM cell entry into the bone marrow microenvironment [73,74].

The Wnt/β-catenin signaling pathway is aberrantly activated in MM, promoting cell proliferation. In this pathway, Wnt ligands bind to Frizzled and LRP5/6 receptors, leading to inhibition of the β-catenin destruction complex (comprising GSK-3β, APC, and Axin). This inhibition stabilizes β-catenin, allowing its nuclear translocation and interaction with TCF/LEF transcription factors, which activates the genes involved in proliferation, survival, and migration. Notably, Wnt signaling upregulates oncogenes such as *CCND1* and *MYC*, while downregulating regulators like Aurora Kinase A (*AURKA*) [75]. BMECs also express BCL9, a transcriptional co-activator involved in the hyperactivation of Wnt/β-catenin signaling in MM [76].

Interestingly, CyPA has been shown to interact with β-catenin, potentially influencing the transcription of Wnt target genes, including epithelial–mesenchymal transition (EMT) markers such as Snail (*SNAI1*) and Vimentin (*VIM*) [77]. Previous studies have shown that, in glioma, CyPA requires binding to β-catenin to translocate into the nucleus, as CyPA fails to localize to the nucleus in cells lacking β-catenin. Moreover, in CyPA-deficient cells, the interaction between β-catenin and TCF4 is reduced, leading to impaired Wnt-related gene transcription, whereas in β-catenin-deficient cells, CyPA is unable to enter the nucleus and interact with TCF4 [78]. Furthermore, CD147 enhances Wnt/β-catenin signaling in prostate cancer by inhibiting GSK-3β phosphorylation activity (via Ser9), degradation of β-catenin, and promoting EMT [79]. However, further studies are needed to validate this axis in MM.

Sialylation plays a key role in regulating E- and P-selectin ligands on BMECs, which mediate MM cell adhesion and transendothelial migration. Overexpression of the sialyltransferase (*ST3Gal-6*) enhances MM homing to the bone marrow, whereas its knockdown impairs this process [80,81]. Targeting sialylation may reduce MM cell entry into the protective bone marrow niche, where they typically evade chemotherapy, including agents like bortezomib.

E-selectin, expressed on BMECs, supports MM cell homing and bone metastasis [74], while P-selectin, found on both BMECs and BMSCs, facilitates MM–platelet interactions, aiding immune evasion and metastasis [82]. MM cells express high levels of P-selectin glycoprotein ligand-1 (PSGL-1), which has greater affinity for P-selectin than E-selectin. PSGL-1 promotes MM cell adhesion, homing, survival, and drug resistance via E- and P-selectin engagement [83]. The PSGL-1/P-selectin interaction activates downstream pathways such as FAK, Src, and PI3K/AKT, promoting adhesion, migration, proliferation, and survival [84,85,86]. PSGL-1 engagement by E-selectin also activates Src family kinases like Fgr, leading to the phosphorylation of p38 MAPK and the recruitment/activation of spleen tyrosine kinase (Syk) [87,88,89].

Sialylation of integrin subunits α4β1 and α4β7—both highly expressed on MM cells—enhances interactions with VCAM-1 and mucosal vascular addressin cell adhesion molecule 1 (MadCAM-1), contributing to MM homing [90].

VCAM-1, expressed on BMECs and BMSCs, binds to MM cell surface integrins (e.g., VLA-4/α4β1), CD44, and CD56, promoting cell adhesion, proliferation, and resistance to apoptosis [91,92,93]. MadCAM-1 (BMEC-expressed) and ICAM-1 (BMSC-expressed) interact with α4β7 and LFA-1 (αLβ2), respectively, further supporting MM cell retention in the bone marrow. These adhesion events activate the NF-κB pathway, driving the expression of cytokines (e.g., IL-6), chemokines (e.g., CCL2), anti-apoptotic proteins (e.g., Bcl-2, Bcl-xL), and adhesion molecules (e.g., VCAM-1). This signaling cascade contributes to cell-adhesion-mediated drug resistance (CAM-DR), enhancing MM survival and homing [22,94,95].

Finally, stromal-cell-derived factor 1α (SDF-1α/CXCL12), highly expressed by BMSCs and BMECs, binds to CXCR4 on MM cells, promoting their migration into the bone marrow [96]. CXCL12/CXCR4 interaction upregulates integrins VLA-4 and LFA-1, strengthening MM cell adhesion to VCAM-1 and ICAM-1, respectively [96,97]. CXCR4 seems to play a role in the acquisition of EMT phenotype, leading to MM migration and metastasis [98]. CXCR4-CXCL12 axis can activate p-ERK and p-AKT, leading to PI3K/AKT and ERK pathways activation, which are associated with the homing, survival, proliferation, and migration of MM cells [98,99,100].

**Figure 1:**
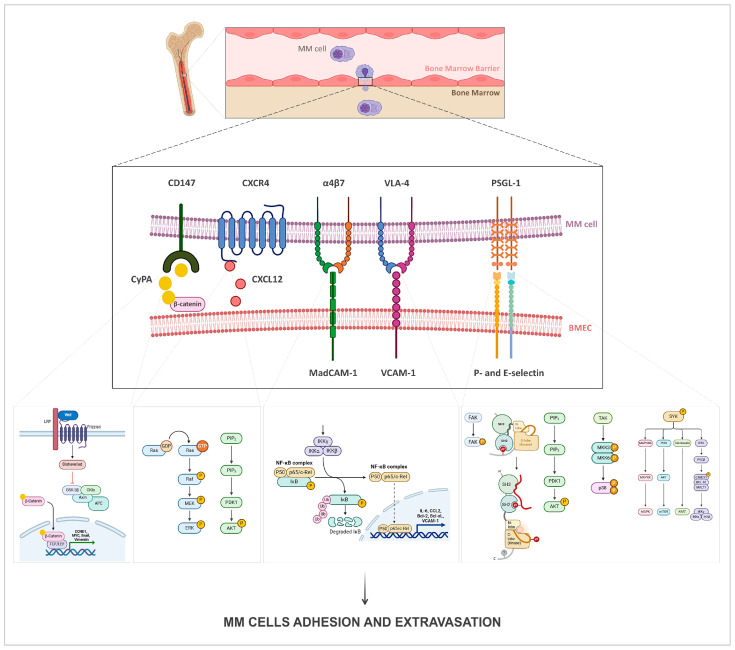
Key signaling pathways in multiple myeloma cell adhesion and extravasation into the bone marrow microenvironment. Surface molecules expressed on multiple myeloma (MM) cells and their corresponding ligands or receptors on bone marrow endothelial cells (BMECs) mediate critical signaling pathways that facilitate MM cell trafficking, adhesion, and survival. CD147 on MM cells binds extracellular cyclophilin A (CyPA), which interacts with β-catenin and promotes its association with TCF/LEF, leading to the transcriptional activation of *CCND1*, *MYC*, *SNAI1*, and *VIM*, thereby enhancing MM cell survival and adhesion. The chemokine receptor CXCR4 binds its ligand CXCL12, driving MM cell migration toward the bone marrow niche and activating p-ERK and p-AKT signaling pathways. Integrins α4β7 and VLA-4 on MM cells engage with MadCAM-1 and VCAM-1 on BMECs, respectively, triggering NF-κB activation and the subsequent upregulation of cytokines (IL-6), chemokines (CCL2), anti-apoptotic proteins (Bcl-2, Bcl-xL), and additional adhesion molecules (VCAM-1). PSGL-1 on MM cells interacts with P- and E-selectins on BMECs, mediating rolling, adhesion, and transendothelial migration, through phosphorylation of FAK, Src kinase, AKT, p38, and Syk. The Figure was generated using BioRender.com.

These signaling events involved in MM cell homing and bone marrow colonization are summarized in Table 1 and illustrated in Figure 1.

### 4.2. Proliferation Pathways

Peroxisome proliferator-activated receptor β/δ (PPARβ/δ) is upregulated in various tumors and is associated with cancer progression, relapse, and metastasis. PPARβ/δ is expressed in endothelial cells, and its activation by prostacyclin I2 (PGI2) promotes the expression of VEGF and its receptor, both crucial for neovascularization. This activation enhances endothelial cell migration and proliferation [101,102,103]. However, the correlation between PPARβ/δ and MM remains poorly understood. A recent study suggests that PPARβ/δ expression is higher in BMECs of MM patients compared to those with monoclonal gammopathy of undetermined significance (MGUS) [104]. MM cells stimulate PGI2 release, which, in turn, activates PPARβ/δ, promoting angiogenesis and MM progression. The inhibition of PPARβ/δ disrupts the angiogenic capability of MM-associated vessels [104].

Epidermal growth factor receptor (EGFR) and heparin-binding EGF-like growth factor (HB-EGF) are overexpressed in the BMECs of MM patients compared to MGUS. HB-EGF stimulates EGFR expression via an autocrine loop, leading to angiogenesis. Blocking HB-EGF has been shown to impair neovascularization. Therefore, HB-EGF–EGFR signaling may play a role in the transition from the avascular to the vascular phase of MM, contributing to disease progression [105]. However, the role of HB-EGF–EGFR signaling in hematological cancers remains underexplored and warrants further investigation.

The adhesion of MM cells to BMSCs promotes the secretion of VEGF, IL-6, insulin-like growth factor 1 (IGF-1), and other growth factors [97]. These cytokines support MM cell growth and metastasis by activating key proliferative and anti-apoptotic pathways, including JAK/STAT, Ras/Raf/MEK/MAPK, and PI3K/Akt/mTOR [106,107,108].

JAK2 is highly expressed in MM cells. IL-6 binding to its receptor activates JAK2, which phosphorylates STAT3. Phosphorylated STAT3 translocates to the nucleus and promotes the transcription of pro-survival genes, including *Bcl-xL* and *Mcl-1* [94,109,110]. IL-6 is thus a central mediator of MM progression and also activates the Ras/Raf/MEK/MAPK and PI3K/Akt/mTOR pathways [111]. VEGF, an angiogenic cytokine, binds VEGFR-2 on endothelial cells, activating PI3K/Akt and Raf/MEK/ERK pathways, thereby promoting endothelial migration, and MM cell survival and proliferation [112]. IL-6 and VEGF enhance the phosphorylation of ERK1/2 and Ras activation, leading to the expression of oncogenic transcription factors like *MAF*, which is associated with MM cell proliferation and drug resistance [94,113]. The activation of PI3K by IL-6, VEGF, and IGF-1 leads to Akt phosphorylation, enhancing proliferation and anti-apoptotic responses through both mTOR-dependent and mTOR-independent mechanisms [114].

Erythropoietin (Epo) is a cytokine that exerts hematopoietic and extra-hematopoietic functions. Its receptor (EpoR) is more highly expressed by BMECs in MGUS. The interaction between Epo and EpoR activates angiogenic effects via PI3K/Akt and JAK/STAT pathways in MGUS, promoting endothelial cell migration. EpoR also increases the phosphorylation of JAK2, STAT5, and Akt, and regulates the secretion of pro-angiogenic factors, including VEGF, thereby contributing to tumor growth [115,116,117].

IGF-1 is a proliferative and anti-apoptotic factor that induces the EMT phenotype in various cancers through activation of signaling pathways, particularly PI3K/Akt. Although little is known about IGF-1-mediated EMT in MM metastasis, a recent study showed that IGF-1 plays a critical role in the migration and invasion of MM cells from the bone marrow microenvironment by inducing EMT via PI3K/Akt signaling [118]. Increased IGF-1 expression correlates with elevated levels of EMT markers, such as N-cadherin, Vimentin, and Slug. IGF-1 enhances Akt phosphorylation, thereby confirming activation of the PI3K/Akt pathway. Notably, Peng et al. demonstrated that the inhibition of PI3K/Akt signaling using an Akt inhibitor significantly reduced EMT marker expression.

These signaling events involved in MM cell proliferation are summarized in Table 2.

## 5. Diagnosis and Treatments of Multiple Myeloma

Accurate diagnosis and disease monitoring are critical for guiding therapeutic approaches in MM. In recent years, the evaluation of minimal residual disease (MRD) has emerged as a strong predictor of progression-free and overall survival, and it is increasingly used to assess the effectiveness of novel therapies. While bone marrow aspirates and biopsies remain the gold standard for MM diagnosis, recent approaches are shifting toward peripheral blood as a less invasive alternative. MRD monitoring through next-generation sequencing (NGS) and multiparameter flow cytometry (MFC) in peripheral blood can reflect systemic disease burden [119]. Peripheral-blood-based diagnostic strategies are also currently being explored in AML, with growing interest in developing standardized and minimally invasive procedures [120]. Emerging evidence supports the detection of changes in the expression of adhesion molecules and chemokine receptors on circulating MM cells in peripheral blood. Differences in the expression of these biomarkers between bone marrow and peripheral blood suggest that their evaluation could represent a predictive tool for extramedullary dissemination [121]. In addition, extracellular vesicles (EVs), released by MM cells and BMSCs, can encapsulate microRNAs (miRNA) and contribute to drug resistance, immune evasion, and disease progression [122]. In the future, MRD assessment models could be integrated with biomarkers (including EVs) that reflect microenvironment-related factors, thereby enhancing the precision of disease monitoring and providing additional insight into treatment response and relapse risk.

Over the past two decades, treatment strategies for MM have significantly evolved. Among the conventional non-targeted therapies, proteasome inhibitors (bortezomib), histone deacetylase inhibitors (panobinostat), and immunomodulatory agents (lenalidomide and pomalidomide) have shown efficacy in delaying MM progression.

More recently, targeted therapies have revolutionized the therapeutic landscape, providing more precise approaches and improving outcomes. These include monoclonal antibodies, such as anti-CD38 (aratumumab, isatuximab) and anti-SLAMF7 (elotuzumab), immune checkpoint inhibitors targeting PD-1/PD-L1 (e.g., nivolumab, pembrolizumab), and chimeric antigen receptor-T (CAR-T) [123]. In addition, nuclear export inhibitors (selinexor) and CAR-T cells targeting GPRC5D or BCMA have shown encouraging results in relapsed or refractory disease [124]. Continued research and clinical trials are essential to expand treatment options, focusing on signaling pathways involved in MM cell homing and proliferation that may provide insights for novel therapeutic strategies.

Building upon these advances, a newer class of immunotherapies known as bi-specific antibodies (BsAbs) has emerged, offering distinct advantages over existing modalities. Unlike CAR-T therapies, which require a complex and time-consuming manufacturing process associated with a high risk of disease progression, BsAbs offer greater availability and reliability. Moreover, they also tend to be associated with a lower risk of immune-related side effects and neurotoxicity, and may represent a more effective option for patients with relapsed or refractory MM [125].

BsAbs are composed of two binding sites and can interact with both an immune effector cell and a tumor cell. BsAbs can minimize toxicity by targeting highly specific antigens on MM cells, such as BCMA, GPRC5D, and Fc recep-tor–homolog 5 (FcRH5), and have demonstrated efficacy in relapsed and refractory MM [126].

Among BsAbs, bi-specific T-cell engagers (BiTEs) represent a particularly effective subclass. These molecules bind CD3 on T cells and redirect cytotoxic activity toward MM cells. Notably, the European Medicines Agency (EMA) has approved teclistamab and elranatamab, which target BCMA, as well as talquetamab, which targets GPRC5D, for use in relapsed and refractory settings [127].

Ongoing clinical trials are further expanding this class, investigating BsAbs such as linvoseltamab (anti-BCMA) and cevostamab (anti-FcRH5), including combination strategies with other BsAbs or anti-MM agents. These studies aim to explore synergistic effects, enhance response durability, and broaden treatment options for patients with heavily pretreated MM [128].

Continued research and clinical trials are essential to expand treatment options, focusing on signaling pathways involved in MM cell homing and proliferation that may provide insights for novel therapeutic strategies.

## 6. Conclusions

Despite significant advances in the treatment of MM, including the development of immunotherapies such as CAR-T cells and bispecific antibodies, the disease remains incurable, and most patients eventually relapse. This review underscores the central role of the bone marrow microenvironment, particularly the BMB in MM progression, highlighting how interactions between MM cells, BMECs, and BMSCs contribute to tumor survival, proliferation, and therapy resistance.

We examined key adhesion molecules and homing and proliferative signaling pathways, which facilitate MM cell trafficking, retention in the marrow niche, and evasion of therapeutic pressure. While many of these pathways are mechanistically well-characterized, they are not yet widely targeted in clinical practice, partly due to the complex and redundant nature of microenvironmental signaling.

Nevertheless, understanding these molecular interactions is essential for identifying combinatorial strategies that may improve long-term disease control. In particular, integrating microenvironment-targeted therapies with existing immunotherapeutic approaches could enhance treatment durability and potentially prevent or delay relapse. Continued research into the MM–bone marrow microenvironment crosstalk will be critical to translating these insights into clinically actionable interventions.

## Figures and Tables

**Table 1:** Homing pathways. Molecules are expressed by bone marrow endothelial cells (BMECs) and bone marrow stromal cells (BMSCs). Their corresponding interaction with multiple myeloma (MM) cells and signaling pathways involved in the homing of MM cells within the bone marrow microenvironment are shown.

Homing	Expressed by	Interaction with	Signaling Pathways	Ref.
CyPA	BMECs	CD147	Wnt/β-catenin	[74,77,78]
P-selectin	BMECs/BMSCs	PSGL-1	FAK, Src and PI3K/AKT	[84,85,86]
E-selectin	BMECs	PSGL-1	Src, p38 MAPK and Syk	[87,88,89]
VCAM-1	BMECs/BMSCs	VLA-4, CD44, CD56	NF-κB	[90,91,92,93]
MadCAM-1	BMECs	α4β7	NF-κB	[97]
ICAM	BMSCs	LFA-1	NF-κB	[95,97]
CXCL12	BMECs/BMSCs	CXCR4	PI3K/Akt and Raf/MEK/ERK	[96,98,99,100]

**Table 2:** Proliferation pathways. Molecules are expressed by bone marrow endothelial cells (BMECs) and bone marrow stromal cells (BMSCs), and their associated signaling pathways involved in multiple myeloma (MM) cell proliferation are shown.

Proliferation	Expressed by	Signaling Pathways	Ref.
BCL9	BMECs	Wnt/β-catenin	[76]
PGI2	BMECs	PPAR β/δ	[104]
HB-EGF	BMECs	HB-EGF-EGFR	[105]
VEGF	BMSCs	PI3K/Akt and Raf/MEK/ERK	[112]
IL-6	BMSCs	JAK/STAT, Ras/Raf/MEK/MAPK and PI3K/Akt/mTOR	[108,109,110,111]
Epo	BMECs	PI3K/Akt and JAK/STAT	[115,116,117]
IGF-1	BMSCs	PI3K/Akt	[118]

## Data Availability

Data are available on request.

## Abbreviations

The following abbreviations are used in this manuscript:

BMB	Bone Marrow Barrier
BMECs	Bone Marrow Endothelial Cells
BMSCs	Bone Marrow Stromal Cells
MM	Multiple Myeloma
AML	Acute Myeloid Leukemia
IL	Interleukin
TNF	Tumor Necrosis Factor
ICAM	Intercellular Cell Adhesion Molecule
VCAM-1	Vascular Cell Adhesion Molecule-1
VLA-4	Very Late Antigen-4
BM	Bone Marrow
VEGF	Vascular Endothelial Growth Factor
Ang	Angiopoietin
NO	Nitric Oxide
iNOS	inducible Nitric Oxide Synthase
eNOS	endothelial Nitric Oxide Synthase
MMPs	Matrix Metalloproteinases
EC	Extracellular Matrix
EMM	Extramedullary Multiple Myeloma
MGUS	Monoclonal Gammopathy of Undetermined Significance
SMM	Smoldering Multiple Myeloma
CyPA	Cyclophilin A
siRNA	Small Interfering RNA
AURKA	Aurora Kinase A
EMT	Epithelial–Mesenchymal Transition
Syk	Spleen Tyrosine Kinase
MadCAM-1	Mucosal Vascular Addressin Cell Adhesion Molecule-1
CAM-DR	Cell-Adhesion-Mediated Drug Resistance
SDF-1α	Stromal-Cell-Derived Factor 1α
PPARβ/δ	Peroxisome Proliferator-Activated Receptor β/δ
PGI2	Prostacyclin I2
EGFR	Epidermal Growth Factor Receptor
HB-EGF	Heparin-Binding EGF-like Growth Factor
IGF-1	Insulin-like Growth Factor-1
Epo	Erythropoietin
MRD	Minimal Residual Disease
NGS	Next-Generation Sequencing
MFC	Multiparameter Flow Cytometry
EVs	Extracellular Vesicles
miRNA	microRNA
CAR-T	Chimeric Antigen Receptor-T
